# Radial artery pseudoaneurysm after a single arterial puncture for blood-gas analysis: a case report

**DOI:** 10.4076/1757-1626-2-6890

**Published:** 2009-07-21

**Authors:** Vincenzo Leone, Daniele Misuri, Nico Console

**Affiliations:** Department of General Surgery, Santa Maria Nuova HospitalASL 10 FirenzeItaly

## Abstract

We report a case of a radial artery pseudoaneurysm complicating a single arterial puncture for blood-gas analysis that was treated with excision of pseudoaneurysm and suture of the defect of wall of radial artery. The puncture for continuous blood pressure monitoring and serial blood gas analysis have been reported in critically ill patients, but, to the best of our knowledge, there are no cases reported of pseudoaneurysm after a single arterial puncture for blood-gas analysis. In the reported case we think that the main cause of the pseudoaneurysm onset was an incorrect compression and/or a too much short time of compression of the radial artery after the puncture. Minor sequelae and rare complications may be minimized by careful attention to detail in the performance of such procedures and care of the patient also after a single arterial puncture.

## Introduction

We report a case of pseudoaneurysm due to a single puncture of radial artery for arterial blood-gas analysis. Only 22 cases of aneurysm of the radial artery have been reported in the literature till 2006 [1]. The vast majority of cases are due to iatrogenic arterial lesions and their incidence has been increasing because of the higher use of interventional radiological procedures such as angioplasty, the positioning of stents, together with the frequent utilization of diagnostic and therapeutic cardiac catheterization. Radial artery catheters inserted for blood pressure monitoring and arterial blood-gas analysis, paravascular infectious, inflammatory, haemodialysis therapy and traumatic processes that disrupt or destroy the arterial wall may also lead to producing arterial wall lesions and consequently a pseudoaneurysm to be created. The puncture for continuous blood pressure monitoring and serial blood gas analysis have been reported in critically ill patients, but, to the best of our knowledge, there are no cases reported of pseudoaneurysm after a single arterial puncture for blood-gas analysis.

## Case presentation

**Figure 1.**
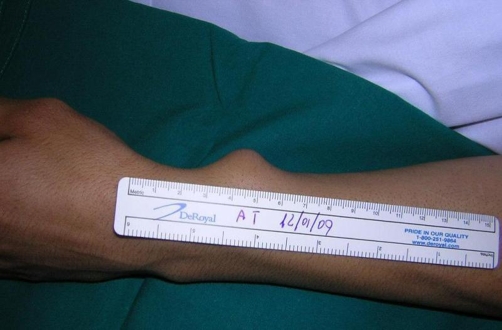
Pulsatile 3 cm mass over the left volar wrist.

**Figure 2.**
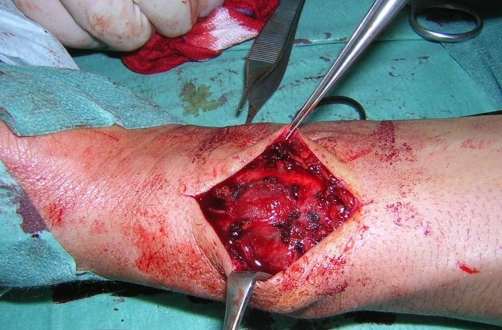
Isolated pseudoaneurysm.

**Figure 3.**
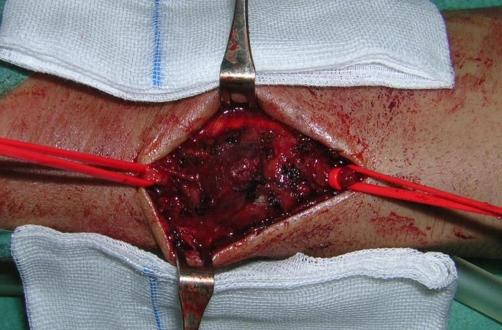
The ends of radial artery were identified and oversaw.

A 32-year-old white man was admitted to Emergency Department for asthma attack. Arterial blood was withdrawn from the left radial artery by direct puncture for arterial blood-gas analysis. The patient was discharged after 10 hours because of the improvement of the respiratory symptoms. Six days later he came back for the onset of a pulsatile 3 cm mass over the left volar wrist (Figure 1). Allen’s test was negative and hand function was normal. The mass was pulsatile and a systolic bruit was audible. There was no palpable temperature disparity between the left and right hands and the patient was afebrile. The patient underwent to colour duplex ultrasound that showed the presence of pseudoaneurysm arising from the main left radial artery, continuous bidirectional blood flow in the neck of the pseudoaneurysm and a turbulent blood flow within the lesion and in the radial artery which was patent. Ulnar artery and palmar arch integrity were also confirmed. Therefore, we decided to perform a surgical exploration of the radial artery under local anesthesia. Both ends of the radial artery were identified and oversaw (Figures 2, 3). A 1-2 millimetre hole in the wall of radial artery communicated with the pseudoaneurysm have been found and sutured with 5/0 prolene. The patient was discharged from the hospital on day 2, with no postoperative complications.

## Discussion

Pseudoaneurysms are usually caused by perforation of an artery with hematoma formation between the arterial wall and surrounding parenchyma. Flowing arterial blood creates a cavity that remains in continuity with the normal vessel and becomes lined by inflammatory cells and fibroblasts and is eventually replaced by fibrous scar tissue. The lesion has a saccular appearance and the false sac is lined with endothelium, and the outer walls are formed of fibrous scar tissue. The pseudoaneurysms of the distal upper extremity may mimic many other soft tissue masses. Therefore, diagnostic puncture of a wrist swelling may be unwise. The real incidence is unknown, probably because these lesions are seldom reported.

Radial artery catheterization is a safe, frequently performed procedure for continuous blood pressure monitoring and serial blood gas analysis, but it is not free of risk. The most common complication is thrombosis. In our reported case the pseudoaneurysm owing to a single puncture of radial artery for arterial blood-gas analysis. This occurrence is a very unusual case because of the vast majority of cases are observed late after catheterization (7-40 days) for continuous blood pressure monitoring and serial blood gas analysis in critically ill patients, and are associated with vessel wall alterations, repeated puncture attempts, and catheter infection [2]. A retrospective analysis of 8300 patients undergoing cardiac surgery showed that the incidence of radial infection is 0.2% - 0.7% with bacteraemia in 0.15% and the development of pseudoaneurysms in 0.05% [3]. Other rare causes to develop a radial artery pseudoaneurysm are described: in haemophiliac patients after radial artery puncture [4] and following inadequate radial fracture stabilization with a plate [5]. Conservative treatment has recently become a reliable alternative to surgical intervention in adults with pseudoaneurysm following iatrogenic injury of the artery. Available non-surgical measures rely on thrombus formation, and include ultrasound guided compression repair, reapplication of a compression bandage, and clinical observation of the natural course. Ultrasound guided compression repair is a non-invasive technique characterised by manual compression of the pseudoaneurysm with the transducer probe, maintained for 10 minute intervals, after which time the lesion is rechecked for occlusion. If flow is still present, compression is quickly re-established for additional 10 minute intervals, until occlusion is achieved. Another non-surgical treatment is the percutaneous thrombin injection under ultrasound guidance which has become the treatment of choice for embolization of iatrogenic femoral pseudoaneurysm, and recently has been performed in arteries in other districts [6]. Surgical intervention is needed in complicated cases (symptomatic, expanding, infection, prolonged history, and with large haematomas), and in patients with failed conservative management. Prompt surgery has been advocated because of the potential risk of rupture and thromboembolism. The options include ligation of the artery if distal circulation is not compromised, excision of the pseudoaneurysm, and anastomosis using patch graft. However, current operative management after excision of radial pseudoaneurysm remains controversial, with some authors advising vascular reconstruction, and others opting for arterial ligation. Proponents of vascular reconstruction cite the possibility of elimination of complications, such as cold and work intolerance [7]. Moreover, the importance of full restoration of the hand blood supply is stressed in children, to prevent retardation of extremities growth [8]. Other authors support simple ligation of radial artery and excision of the lesion if adequate collateral flow can be shown with Allen’s test, colour duplex ultrasound and evidence of backbleeding from the distal stump. However, ligation of the radial artery may lead to digital ischaemia, if the collateral circulation to hand is inadequate. Allen’s test and its modifications are widely used to evaluate the adequacy of the collateral circulation even though this has significant false positive and false negative results. As an alternative, excision of the pseudoaneurysm and patch vein graft gives good results. The patient was operated on in our case with excision of pseudoaneurysm and suture of the defect of wall of radial artery, and follow up at 1 and 3 months showed no recurrence of the pseudoaneurysm with patency of the radial artery. In the reported case we think that the main cause of the pseudoaneurysm onset was an incorrect compression and/or a too much short time of compression of the radial artery after the puncture. Minor sequelae and rare complications may be minimized by careful attention to detail in the performance of such procedures and care of the patient also after a single arterial puncture.
